# ‘With age comes wisdom’: effectiveness and tolerability of dolutegravir + lamivudine in virologically-suppressed people with HIV

**DOI:** 10.1097/QAD.0000000000004153

**Published:** 2025-03-06

**Authors:** Gianmaria Baldin, Pierluigi Francesco Salvo, Rosa Anna Passerotto, Valentina Iannone, Francesco Lamanna, Andrea Carbone, Damiano Farinacci, Francesca Lombardi, Alberto Borghetti, Simona Di Giambenedetto, Carlo Torti, Arturo Ciccullo

**Affiliations:** a Infectious Diseases Unit, Fondazione Policlinico Universitario A. Gemelli IRCCS; b Department of Safety and Bioethics, Catholic University of the Sacred Heart, Rome; c Infectious Diseases Clinic, Azienda Ospedaliero Universitaria Pisana, Pisa; d Infectious Diseases Unit, San Salvatore Hospital, L’Aquila, Italy.

**Keywords:** dolutegravir, effectiveness, HAART, HIV, lamivudine, simplification

## Abstract

**Objectives:**

Results from clinical trials and observational studies suggest that dolutegravir plus lamivudine is a well tolerated option for simplification in people with HIV (PWH). We aimed to assess long-time effectiveness and safety in our cohort.

**Methods:**

We performed an observational study enrolling HIV-1-infected, virologically suppressed PWH, switching to dolutegravir plus lamivudine. Exclusion criteria were HBV-coinfection and the presence of the M184V mutation before the simplification. We performed survival analysis to evaluate time to virological failure (VF, defined by a single HIV-RNA ≥200 copies/ml or by two consecutive HIV-RNA ≥ 50 copies/ml) and treatment discontinuation (TD, defined as the interruption of either 3TC or DTG).

**Results:**

Six hundred thirty-one PWH were considered for the analysis: 446 were males (70.7%), with a median age of 51.1 years [interquartile range (IQR) 42.6–57.6]. Estimated probabilities of maintaining virological suppression at 192 and 384 weeks were 95.1% [95% confidence interval (CI) 92.0–96.2] and 91.5% (95% CI 87.1–94.4), respectively. At multivariable analysis, including zenith HIV-RNA, time of virological suppression before switch, risk factors for HIV infection and age, only intravenous drug users (IDU) [versus other risk factors, adjusted hazard ratio (aHR) 3.58, 95% CI 1.38–9.28, *P* = 0.009] independently predicted VF. A border-line significant association with VF emerged for age (per 10-years more, aHR 0.72, 95% CI 0.51–1.01, *P* = 0.058) and zenith HIV-RNA >500 000 cps/ml (versus. <500 000 cps/ml, aHR 2.31, 95% CI 0.98–5.46, *P* = 0.056).

As to treatment tolerability, estimated probabilities of remaining on study regimen at 192 and 384 weeks were 87.8% (95% CI 84.5–90.5) and 85.1% (95% CI 81.0–88.5), respectively.

**Conclusions:**

Our findings confirm the long-term effectiveness and tolerability of dolutegravir plus lamivudine in virologically suppressed PWH.

## Introduction

HIV-1 infection continues to pose a significant global health challenge, prompting ongoing advancements in therapeutic strategies. Antiretroviral therapy (ART) has emerged as an indispensable tool in the management of the HIV pandemic, substantially improving patient outcomes and enhancing overall quality of life [1]. Traditionally, combination regimens involving three or more drugs have been the primary approach to treatment. However, recent developments mark a notable shift in perspective, with a growing emphasis on two-drug regimens (2DRs) as effective alternatives [2,3].

The TANGO study, conducted among people with HIV (PWH) who have experienced previous treatment, stands out as a pivotal investigation [4]. This study demonstrated that switching to a dual therapy comprising lamivudine (3TC) and dolutegravir (DTG), in contrast to continuing a triple therapy regimen based on tenofovir alafenamide (TAF), was noninferior in terms of virological efficacy. Notably, this switch did not result in any treatment interruptions related to virological failures after 144 weeks of follow-up. These findings hold significant implications, especially considering the evolving landscape of HIV management.

In response to such compelling evidence, contemporary treatment guidelines have incorporated 2DRs, like 3TC/DTG, as first-line therapies for both treatment-naive individuals and those needing therapeutic simplification [5,6]. The strategic move towards 2DRs reflects not only their comparable effectiveness but also the potential for reduced drug-related toxicities and improved adherence [7]. This paradigm shift aligns with the broader goal of optimizing HIV care by tailoring treatment approaches to individual needs.

This study aims to evaluate the viroimmunological effectiveness and durability of 3TC/DTG in a cohort of PWH under our outpatient clinic's care, with an extensive 8-year follow-up period.

## Materials and methods

This was an observational study enrolling HIV-1-infected individuals, virologically suppressed from at least 24 weeks, switching to 3TC/DTG. We retrospectively included adults (age >18 years) PWH starting 3TC/DTG in our clinical center in Rome between January 2015 and February 2023. Exclusion criteria were: HBV-coinfection (defined as HBsAg detection) and the presence of the M184V mutation at any genotypic analysis performed prior to 3TC/DTG initiation. We performed survival analyses to evaluate time to virological failure (VF, defined by a single HIV-RNA ≥200 copies/ml or by two consecutive HIV-RNA ≥ 50 copies/ml) and treatment discontinuation (TD, defined as the interruption of either 3TC or DTG or the intensification of the ART regimen); predictors of VF or TD were analyzed by Cox regression. All covariates with a *P*-value ≤0.05 at the univariate analysis were tested into multivariate regression models, using a backward stepwise selection method. The study was performed according to the principles of the Declaration of Helsinki and received the approval by each independent local Ethics Committee (study coordination site protocol number 5284/15).

## Results

We analyzed data from 631 PWH: 446 were males (70.7%), with a median age of 51.1 years [interquartile range (IQR) 42.6–57.6], a median time from HIV diagnosis of 12.7 years (6.5–19.7) and a median time of ART exposure of 10.2 years (5.6–11–1). The majority of PWH switched to 3TC/DTG due to proactive simplification (62.1%), with almost half of the population coming from a 3-drug regimen with 2 NRTIs and an Integrase Inhibitor (266, 42.3%). Full population characteristics at baseline are presented in Table 1.

**Table 1:** Patients’ characteristics at baseline (*N* = 631).

Variables	
Age (years), median (IQR)	51.1 (42.6–57.6)
Male, *n* (%)	446 (70.7)
Risk factor for HIV infection, *n* (%):	
Heterosexual	261 (41.4)
MSM	264 (41.8)
IDU	45 (7.1)
Others	61 (9.7)
Anti-HCV antibodies positive, *n* (%)	74 (11.8)
Time from HIV diagnosis (years), median (IQR)	12.7 (6.5–19.7)
CDC stage C, *n* (%)	144 (22.8)
Time on antiretroviral therapy (years), Median (IQR)	10.2 (5.6–11.1)
Nadir of CD4^+^ (cell/μl), median (IQR)	220 (82–331)
Zenith HIV-RNA (log copies/ml), median (IQR)	4.91 (4.34–5.42)
Zenith HIV-RNA >500 000 copies/ml, *n* (%)	63 (10.9)
Previous virological failure, *n* (%)	244 (38.7)
CD4^+^ cell count (cell/μl), Median (IQR)	665 (513–841)
Time of virological suppression (months), median (IQR)	92 (48–141)
Previous HAART regimen, *n* (%):	
2NRTIs + NNRTI	170 (27.0)
2NRTIs + PI or b/PI	29 (4.6)
2NRTIs + INI	266 (42.3)
3TC + b/PI	135 (21.5)
Other	29 (4.6)
DTG in previous regimen, *n* (%)	205 (32.6)
Reasons for switch, *n* (%):	
Simplification	392 (62.1)
Dyslipidemia	64 (10.1)
Gastrointestinal or liver toxicity	21 (3.3)
Renal toxicity	14 (2.2)
Neurological toxicity	5 (0.8)
Other toxicities	38 (6.0)
Drug–drug interactions	9 (1.4)
Other/unknown reasons	88 (13.9)

3TC, lamivudine; DTG, dolutegravir; IDU, intravenous drug users; IQR, interquartile range; MSM, men who have sex with men; NNRTI, non nucleoside reverse transcriptase inhibitors; PI, protease inhibitor.

During 2077.7 person-year of follow-up (PYFU), we registered 31 VF, a rate of 1.5 per 100 PYFU; we did not observe any newly acquired resistance mutation to either 3TC or DTG at a subsequent genotypic analysis in PWH experiencing VF. Estimated probabilities of maintaining virological suppression at 48, 144 and 384 weeks were 98.5% [95% confidence interval (CI) 98.0–99.0], 95.1% (95% CI 92.0–96.2) and 91.5% (95% CI 87.1–94.4), respectively. At multivariable analysis, including zenith HIV-RNA, time of virological suppression before switch, risk factors for HIV infection and age, only having intravenous drug users (IDU) as a risk factor for HIV [versus. other risk factors, adjusted hazard ratio (aHR) 3.58, 95% CI 1.38–9.28, *P* = 0.009] independently predicted VF. A border-line significant association with VF emerged for age (per 10 years more, aHR 0.72, 95% CI 0.51–1.01, *P* = 0.058) and zenith HIV-RNA >500 000 cps/mL (versus. <500 000 cps/ml, aHR 2.31, 95% CI 0.98–5.46, *P* = 0.056). Results from the multivariate analysis are expressed in Table 2.

**Table 2:** Multivariate regression analyses researching predictors of VF.

		95% Confidence interval		
Variable	aHR	Upper limit	Lower limit	*P*
Age	0.72	0.51	1.01	0.058
Time of virological suppression	0.99	0.98	1.01	0.227
Peak HIV-RNA over 500 000 cps/ml	2.31	0.98	5.46	0.056
HIV risk-factor: IDU	3.58	1.38	9.28	0.009

aHR, adjusted hazard ratio; IDU, intravenous drug users.

In our cohort, during 2111.1 PYFU, we observed 67 discontinuations, a rate of 3.2 TD per 100 PYFU. Reasons for TD were: toxicity in 28 cases (41.8% of all discontinuations), switch to a Single Tablet Regimen (7, 10.5%), virological failure in three cases (4.5%), pregnancy (2, 3.0%), other or unknown reasons (28, 41.8%). Further analyzing causes reported as toxicity, we found 10 TD due to neurological toxicity, 7 due to gastro-intestinal toxicity, 2 due to hypersensitivity reactions and 9 due to other/unspecified toxicities.

Estimated probabilities of remaining on study regimen at 48, 144 and 348 weeks were 95.0% (95% CI 94.1–95.9), 87.8% (95% CI 84.5–90.5) and 85.1% (95% CI 81.0–88.5), respectively. We did not find any predictor of TD in our multivariate regression analyses.

## Discussion

Our results show an overall high effectiveness and safety of DTG + 3TC as a switch strategy, even in the long term. In our analysis, we found that over 91% of PWH maintained virological suppression after 384 weeks of follow-up, a follow-up time not previously described in literature and not yet reached by clinical trials [4,8].

Moreover, we confirm previous data from our cohort [9] regarding the genetic barrier of the regimen, with no new resistance mutation observed in those PWH who experience VF.

In our analysis, we found, although not significant, that there is a possible association between peak HIV-RNA and VF, a finding already observed in our cohort [10], that may be due to the impact of HIV reservoir on virological response to the analyzed 2DR in some PWH.

Regarding treatment tolerability, we confirm the excellent tolerability of DTG + 3TC as a switch strategy, with over 85% of analyzed PWH maintaining the study regimen after 384 weeks of follow-up. It is worth noting that given the long follow-up time, some PWH started this regimen as a two-pill regimen, before the introduction of the STR solution. Thus, the seven discontinuations due to switch to a STR, may not have happened with the availability of DTG/3TC as a STR. Although the follow-up time in our cohort is much longer, overall discontinuation rates appear to be lower than those presented in published studies from other real-life cohorts [11–13]. For reference, in the work by Rocabert *et al.*[12], TD rate was 8.44 per 100 PYFU after a median follow-up of 52 weeks, compared to our rate of 3.2 per 100 PYFU. In the study by Maggiolo *et al.*[13], after 240 weeks of follow-up, 77.1% of the cohort maintained DTG+3TC, compared to the estimated probability of maintaining study regimen of 85.1% at 384 weeks observed in our study.

In our work, 28 PWH discontinued the regimen due to toxicity, accounting for 4.4% of overall population, a rate of TD due to toxicity in line with published literature [7].

Our work presents some limitations, such as its retrospective nature and the lack of data regarding low-grade toxicities not causing treatment discontinuations; however, to our knowledge, our results present effectiveness and safety data on DTG + 3TC with the longest follow-up time published to this day.

In conclusion, our work confirms the high effectiveness and overall good tolerability of DTG+3TC as a switch strategy, with promising results even after 8 years of follow-up, thus giving helpful information to clinicians regarding real-life, long-term data on this 2-drug switch strategy.

## Acknowledgements

G.B., A.B., S.D.G. and A.Ci conceived the work, performed statistical analysis and wrote the manuscript. CT supervised and revised the manuscript. P.F.S., R.A.P., V.I., F.La, A.Ca, D.F. and F.Lo collected data.

Funding: We performed this work as part of our routine clinical practice.

### Conflicts of interest

A.Ci received fees from ViiV Healthcare and MSD. A.B. has received nonfinancial support from Bristol-Myers Squibb and ViiV Healthcare, and personal fees from Gilead Sciences and Janssen. S.D. G. was a paid consultant or member of advisory boards for Gilead, ViiV Healthcare, Janssen-Cilag, Merck Sharp & Dohme and Bristol-Myers Squibb. All other authors: none to declare.
